# Yerba Mate (*Ilex paraguariensis*) Reduces Colitis Severity by Promoting Anti-Inflammatory Macrophage Polarization

**DOI:** 10.3390/nu16111616

**Published:** 2024-05-25

**Authors:** Alexandra Olate-Briones, Sofía Albornoz-Muñoz, Francisca Rodríguez-Arriaza, Valentina Rodríguez-Vergara, Juan Machuca Aguirre, Chaohong Liu, Carlos Peña-Farfal, Noelia Escobedo, Andrés A. Herrada

**Affiliations:** 1 Lymphatic Vasculature and Inflammation Research Laboratory, Instituto de Ciencias Biomédicas, Facultad de Ciencias de la Salud, Universidad Autónoma de Chile, Talca 3460000, Chile; alexandra.olate@uautonoma.cl (A.O.-B.); sofia.albornoz@alu.ucm.cl (S.A.-M.); franciscarodriguezarriaza@gmail.com (F.R.-A.); valentina.rodriguez.vergara@gmail.com (V.R.-V.); 2 Investigación y Desarrollo Tecnológico Temuco (IDETECO), Instituto de Ciencias Aplicadas, Facultad de Ingeniería, Universidad Autónoma de Chile, Av. Alemania 01090, Temuco 4810101, Chile; juan.machuca@uautonoma.cl (J.M.A.); carlos.pena@uautonoma.cl (C.P.-F.); 3 Department of Pathogen Biology, School of Basic Medicine, Tongji Medical College, Huazhong University of Science and Technology, Wuhan 430074, China; chaohongliu@hust.edu.cn

**Keywords:** Yerba Mate, DSS-induced colitis, macrophages, inflammation

## Abstract

Yerba Mate (YM) (*Ilex paraguariensis*) is a natural herbal supplement with a well-described anti-inflammatory capacity and beneficial effects in different inflammatory contexts such as insulin resistance or obesity. However, whether YM could improve other inflammatory conditions such as colitis or the immune cell population that can be modulated by this plant remains elusive. Here, by using 61 male and female C57BL/6/J wild-type (*WT*) mice and the dextran sodium sulfate (DSS)-induced acute colitis model, we evaluated the effect of YM on colitis symptoms and macrophage polarization. Our results showed that the oral administration of YM reduces colitis symptoms and improves animal survival. Increasing infiltration of anti-inflammatory M2 macrophage was observed in the colon of the mice treated with YM. Accordingly, YM promoted M2 macrophage differentiation in vivo. However, the direct administration of YM to bone marrow-derived macrophages did not increase anti-inflammatory polarization, suggesting that YM, through an indirect mechanism, is able to skew the M1/M2 ratio. Moreover, YM consumption reduced the *Eubacterium rectale*/*Clostridium coccoides* and *Enterobacteriaceae* groups and increased the *Lactobacillus*/*Lactococcus* group in the gut microbiota. In summary, we show that YM promotes an immunosuppressive environment by enhancing anti-inflammatory M2 macrophage differentiation, reducing colitis symptoms, and suggesting that YM consumption may be a good cost-effective treatment for ulcerative colitis.

## 1. Introduction

Yerba Mate (YM) is a popular tea beverage processed from the leaves and stems of the shrub *Ilex paraguariensis*, widely consumed in Argentina, Brazil, Chile, Paraguay, and Uruguay. YM is traditionally consumed as a hot or cold infusion. It has been recognized for a variety of beneficial biological properties such as antioxidant capacity [1,2], vasodilatation activity, [3], and anti-inflammatory capacity [4,5], among others. These potential therapeutic effects of YM have been attributed to its bioactive compounds, such as polyphenols (chlorogenic acid and tannins), xanthines (caffeine and theobromine), purine alkaloids (caffeic acid, 3,4-dicaffeoylquinic acid, and 3,5-dicaffeoylquinic acid), flavonoids (quercetin, kaempferol, and rutin), saponins, amino acids, minerals (phosphorous, iron, and calcium), and vitamins (C, B1, and B2) [6,7,8].

Inflammatory bowel disease (IBD) is a chronic and relapsing inflammatory disorder of the intestinal tract that encompasses Crohn’s disease and ulcerative colitis [9]. Although the etiology of IBD remains unclear, the combined effects of genetic, microbial, immune, and environmental factors appear to trigger an abnormal and excessive immunological response [10,11]. IBD is characterized by chronic, relapsing and remitting episodes of inflammation of the gastrointestinal tract caused by an abnormal immune response [9]. The most widely used mouse model for ulcerative colitis employs dextran sodium sulfate (DSS), because it reproduces some of the key features of human ulcerative colitis (inflammation, diarrhea, and abnormal feces), making it an excellent model for evaluating possible treatments for this disease [12]. In recent years, it has been shown that macrophages are essential for maintaining intestinal homeostasis as well as inducing inflammation [13,14]. In fact, it is known that during ulcerative colitis, massive infiltration of activated macrophages into the intestinal mucosa occurs, which are phenotypically different from the resident macrophages [15]. Macrophages phagocytose and digest foreign substances and can be found in several tissues, including the epithelial layers of the small intestine and colon, where they regulate steady-state homeostasis and intestinal inflammation [16]. Macrophages exhibit polarized phenotypes that are broadly divided into two categories. Classically activated macrophages, or M1, contribute to the pathogenesis of the disease by secreting proinflammatory cytokines and causing tissue damage, whereas alternatively activated macrophages, or M2, secrete anti-inflammatory factors, playing a key role in the resolution of inflammation [17,18,19]. During DSS-induced colitis, proinflammatory macrophages increase, promoting the production of interleukin (IL)-23 and tumor necrosis factor α (TNFα, while anti-inflammatory macrophages, as well as IL-10 induction, decrease [20]. Since macrophages have opposing roles in colitis, an imbalance of intestinal macrophage populations could modify the course of inflammation. In fact, the regulation of the M1/M2 balance has recently been considered as a potential therapeutic strategy for IBD [21,22]. Currently, there are several treatments for ulcerative colitis such as pharmacological intervention, immunomodulators, gut microbiota modulation, and biological targeting [23,24,25,26,27]. However, these treatments are not effective in all patients; some are expensive and with diverse side effects [28,29].

It is important to highlight that the prevalence of IBD, which translates into a high burden of this inflammatory disease, is markedly higher in industrialized and affluent regions like North America, Europe, Australia, and New Zealand [30,31,32,33]. In contrast, South America has a lower prevalence of IBD compared to the above-mentioned regions [30,34]. Among the countries with the lowest prevalence are Chile, Argentina, Brazil, and Uruguay [34]. These countries are also the main consumers of YM; Uruguay stands out with an annual per capita consumption that exceeds 8 kg, followed by Argentina, where each person consumes an average of 6.5 kg per year [35].

Finding new therapeutic approaches to treat colitis is important because of the need for inexpensive and easily accessible plant-based products, especially in low-income regions where healthcare budgets are limited. The aim of this study was to evaluate the potential immunomodulatory effect of YM consumption on the development of DSS-induced colitis in mice. Macrophages are important mediators of damage in DSS-induced colitis; thus, the possible effect of YM over this innate immune cell type was also evaluated. Finally, we examined the relationship between YM consumption and changes in gut microbiota.

## 2. Materials and Methods

### 2.1. Mice

**Figure 1:**
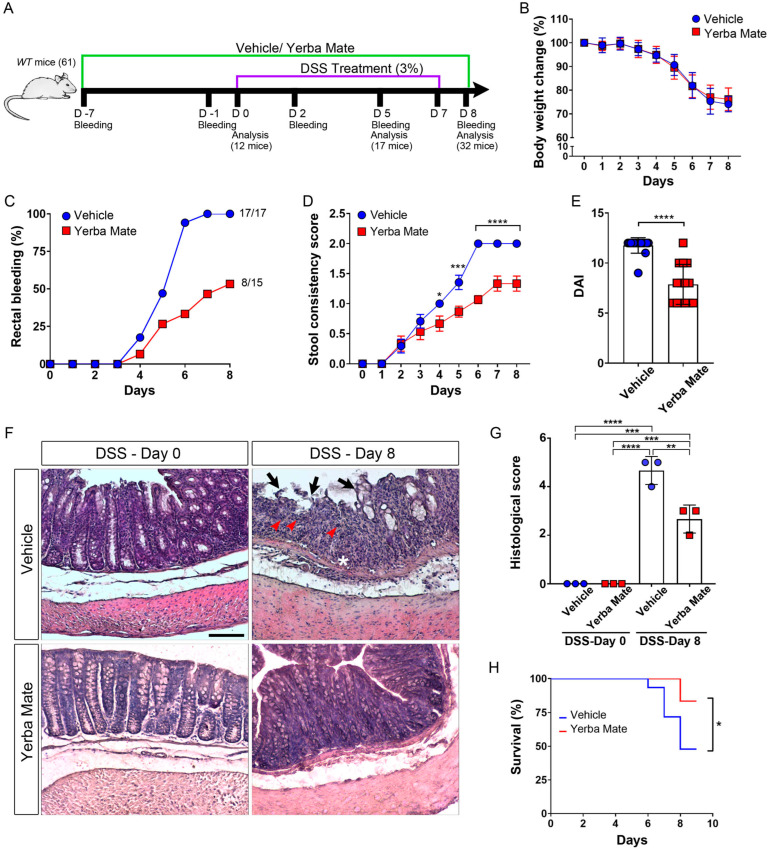
Yerba Mate reduces symptoms and improves survival in a DSS-induced colitis model: (**A**) Flow diagram of the experimental protocol. *WT* mice received YM (0.025 g per mouse) or vehicle by oral gavage seven days before colitis induction, which continued during the entire procedure. The animals were then treated with 3% (*w*/*v*) DSS in the drinking water for 7 days, and the weight was analyzed until Day 8. Serum samples and colon explants were obtained on the indicated days. (**B**) Mouse body weight changes during DSS treatment. Body weight changes were calculated as a percentage of weight prior to DSS treatment. (**C**) Daily rectal bleeding percentage and (**D**) stool consistency score of DSS-induced colitis animals receiving YM or vehicle. (**E**) Disease activity index (DAI) was evaluated as the average score of clinical parameters such as body weight loss, stool consistency, and stool bleeding. Blue circles: vehicle; red squares: yerba mate. (**F**) Histological analysis of medial colon sections of mice treated with Yerba Mate or vehicle followed by DSS treatment. Colons were extracted at Day 0 and Day 8 after DSS treatment, separated into the proximal, medial, and distal colon, and the paraffin sections were analyzed by hematoxylin–eosin staining. Representative images illustrate epithelial inflammation, including neutrophil infiltration (red arrowheads), epithelial damage (black arrows), and ulceration (asterisk), and the irregular shape of colon mucosa in the colons of DSS-treated mice is reduced by Yerba Mate. Scale bar is 100 μm. (**G**) Histological score of the colons of mice treated with vehicle or Yerba Mate before (Day 0) and after DSS treatment (Day 8). Blue circles: vehicle; red squares: yerba mate. The graph shows the mean value ± SD from the analysis of 3 animals per group. (**H**) Survival rate in both mice groups after DSS treatment. Data are plotted as means ± SD from 17 (vehicle group) and 15 (YM group) mice from three independent experiments. * *p* < 0.05; *** *p* < 0.001; **** *p* < 0.0001 by two-way ANOVA followed by Sidak’s multiple-comparison test (**D**), **** *p* < 0.0001 by unpaired *t*-test (**E**), ** *p* < 0.01; *** *p* < 0.001; **** *p* < 0.0001 by one-way ANOVA test followed by Tukey’s multiple-comparison test (**G**) and * *p* < 0.05 by log-rank (Mantel–Cox) test (**H**).

Eight- to twelve-week-old male and female C57BL/6/J wild-type (*WT*) mice, purchased from Jackson Laboratories, were used in this study, and littermates were divided into two groups (control and YM) [36]. To calculate the sample size, the online software of the Chinese University of Hong Kong was used (http://www.lasec.cuhk.edu.hk/sample-size-calculation.html, accessed on 7 July 2020). Thus, a total of 61 mice (44 males and 17 females, since we did not find differences between male and female mice) were used in 3 independent experiments, with 32 mice belonging to the control group and 29 to the YM group. 12, 17, and 32 mice were sacrificed on 0, 5, and 8 days after DSS treatment, and different histological and immune analyses were performed accordingly (which are schematized in Figure 1A). Animals were housed in temperature- and humidity-controlled rooms, maintained on a 12 h light/12 h dark cycle (lights on at 7:00 h), with environmental enrichment, and received water and food ad libitum. All animal procedures and experiments were performed according to the animal protocols approved by the Institutional Animal Care and Use Committee at Universidad Autónoma de Chile (protocol codes BE04-20 and BE01-23). Animals were maintained according to the “Guide to Care and Use of Experimental Animals, Canadian Council on Animal Care”. Inclusion and exclusion criteria were not used in this study.

### 2.2. Yerba Mate Preparation

YM solution was prepared daily by dissolving *Ilex paraguariensis* leaf dry extract (Pajaritos, Paraguay) in hot water at 60 °C and using a homogenizer. The product is intended for human consumption, and it is registered with the appropriate regulatory organization, ensuring compliance with the officially established Good Manufacturing Practices and Production Quality Standards, including the absence or minimum acceptable levels of contaminants. The solution was filtered using a 0.2 µm filter (Thermo Scientific, Waltham, MA, USA) before adding it to the cell culture. For in vitro studies, different concentrations of YM or vehicle were used (1, 10, 25, 50, and 100 µg/mL). For in vivo experiments, 1 g of the leaf dry extract (Pajaritos, Paraguay) was dissolved daily in 8 mL of hot water (60 °C), and the mixture was allowed to cool and then filtered and administered by oral gavage (0.025 g per mouse) daily for seven days before colitis induction, which continued during the entire procedure. Control mice received only the vehicle (hot, chilled, and filtered water). In all the cases, the YM solution (and vehicle) were prepared fresh daily to avoid losing its properties.

### 2.3. Chemical Analysis of Yerba Mate

#### 2.3.1. Chemicals

Acetonitrile and methanol, purchased from Sigma-Aldrich (St. Louis, MO, USA), were of HPLC grade. H_3_PO_4_, >85% was purchased from Sigma-Aldrich (St. Louis, MO, USA) and was of analytical grade. Milli-Q water was obtained from the Milli-Q System. NH_4_H_2_PO_4_ was purchased from Sigma. Chlorogenic acid, quercetin, rutin, quinic acid, theobromine, and caffeine were purchased from Merck (Sigma, St. Louis, MO, USA). Stock solutions at a concentration of 100 mg/L for each analyte were prepared by dissolving a suitable amount of the solid compounds in ethanol. A C-18 reverse-phase column (Kromasil, Shanghai, China) 250 mm l., 4,6 mm i.d. 3.5 dp, was employed for the separation of compounds.

#### 2.3.2. Equipment

A JASCO LC-4000 Series (JASCO, Easton, MD, USA) system with a diode array detector was used for the separation and quantification of compounds of interest. Data acquisition and integration were performed using ChromNAV 2.0 LC software. A 20-microliter injection loop was used. For the determination of the analytes, gradient liquid chromatography was performed as previously described [37], with some modifications. Briefly, the column was thermostated at 35 °C. The total time of the chromatographic analysis was 30 min, and a gradient was used at a flow of 0.4 mL/min. The different phenolic compounds analyzed were identified according to the retention times of pure compounds. The chromatographic recording was carried out at a wavelength of 280 nm.

#### 2.3.3. Analysis of Extracts Compounds

Briefly, 1 g of yerba mate was dissolved by stirring in 8 mL of water at 60 °C, and the mixture was allowed to cool. The sample was filtered through a PTFE filter of 0.22 mm. The standards were also filtered. Calibration curves were plotted between 1 and 10 mg/L. For quantification, the area under each chromatographic peak was calculated. The results are reported as the average of three injections plus minus their standard deviation and expressed as milligrams of analyte per kilogram of Yerba Mate (Supplementary Figure S1). The YM extract contained quinic acid (18 ± 4 mg/Kg), chlorogenic acid (2461 ± 122 mg/Kg), theobromine (892 ± 55 mg/Kg), quercetin (39 ± 7 mg/Kg), rutin (486 ± 16 mg/Kg) and caffeine (2388 ± 189 mg/Kg).

### 2.4. Dextran Sodium Sulfate (DSS) Colitis Model

In order to induce acute colitis in *WT* mice, DSS (3% *w*/*v*) (MP Biomedicals, Irvine, CA, USA) was added to the drinking water for 7 days as previously described [38,39,40,41]. The control group (without DSS) only received water. Monitoring and randomized testing orders on the mice’s general health and condition were conducted daily through a routine measurement of body weight and periodic observation. The severity of colitis was assessed daily using a disease activity index (DAI) that assesses weight loss (1: 1–5%; 2: 5–10%; 3: 10–15%; 4: ≥15%), stool consistency (0: normal; 2: loose stools; 4: diarrhea), and stool blood (0: no blood seen; 2: obvious blood with stool; 4: grossly bloody stool), as previously described [42]. At different time points (Days 0, 5, and 8), anesthetized animals were sacrificed by transcardial perfusion with phosphate-buffered saline 1X (PBS), and the lymph nodes, spleen, and colon were harvested. Tissues were examined for gross macroscopic appearance and stool consistency. The colons were measured, photographed, and analyzed as described below. 

### 2.5. Colon Explants

Colons were isolated from treated mice, washed in PBS with 100 U/mL penicillin G and 100 µg/mL streptomycin (Gibco, Waltham, MA, USA), and weighed; then, 0.5 cm sections were cultured in 500 µL RPMI-1640 (supplemented with 10% fetal bovine serum penicillin, streptomycin, and gentamicin), for 24 h at 37 °C. Supernatants were then collected, and cytokine production was analyzed by ELISA.

### 2.6. Immunohistochemistry

Colon samples were washed with cold PBS and fixed in 4% paraformaldehyde (Sigma-Aldrich) in PBS at 4 °C for 48 h. Samples were then washed in PBS and dehydrated on a sucrose gradient with 15% sucrose in PBS, followed by 30% sucrose overnight at 4 °C. On the next day, tissues were embedded in Tissue-Tek OCT and stored at −80 °C before sectioning. Samples were sectioned at 30 µm with a cryostat and processed for immunofluorescence. Briefly, samples were slightly permeabilized in PBS + 0.5% Triton X-100 for 15 min, blocked with a mixture of 5% donkey serum (Jackson Immuno Research, West Grove, PA, USA) + 1% bovine serum albumin + 0.05% sodium azide and 0.1% Triton X-100 in PBS, and incubated overnight at room temperature with the lymphatic vasculature marker anti-LYVE-1 (Cat No. AF2125, R&D Systems, Minneapolis, MN, USA) at 1:1000 dilution, the macrophage marker anti-F4/80 (Cat No. 35-4801-U100, TONBO, San Diego, CA, USA), and the M2 macrophage marker anti-CD206 (Cat No. 141712, BioLegend, San Diego, CA, USA) at 1:200 dilution. On the next day, samples were washed with PBS + 0.1% Triton X-100 five times for 15 min, and then the samples were incubated with the respective secondary antibodies conjugated with fluorophores at 1:250 dilution for 3 h in a wet chamber. Samples were washed with PBS + 0.1% Triton X-100, mounted with VECTASHIELD antifade mounting medium with DAPI (Vector Laboratories, Newark, CA, USA), and sealed with nail polish. Images were acquired in a confocal microscope Leica Stellaris 5 (Leica Microsystems, Wetzlar, Germany). CD206^+^ cell counts were manually quantified in a blinded manner using ImageJ software (version 1.53t). The number of F4/80^+^CD206^+^ cells was quantified from the photos obtained in the confocal microscope at a magnitude of 63X after performing a z-stack and a maximum projection of the full stack. Cells were quantified by field, and photographs included all the cell layers of the colon (mucosa, submucosa, and muscularis).

### 2.7. Histological Analysis of the Colon

For histopathological analysis, colon samples were collected, washed in PBS, and fixed in 4% paraformaldehyde (Sigma-Aldrich) for 24 h. Then, samples were separated into proximal, medial, and distal samples; dewatered; and embedded in paraffin. Samples were sectioned longitudinally to 8 μm thickness in a microtome (Biobase, Silicon Valley, CA, USA). The sections were dewaxed using Neo-Clear (Sigma, St. Louis, MO, USA), hydrated, and stained with hematoxylin (Sigma-Aldrich) and eosin (Merck). The pathological changes were assessed under an Olympus BX51 microscope (Olympus Life Science, Waltham, MA, USA). The intestinal histological inflammation score was determined as described by Erben et al. (2014) [43]. Briefly, the histopathological score evaluation included the sum of two scores: Score 1 (0–3 points), related to the inflammatory cell infiltration (mucosa; mucosa and submucosa; or transmural), and Score 2 (0–3 points), related to the intestinal architecture (focal erosions; erosions and focal ulcerations; or erosions with extended ulcerations).

### 2.8. Cytokine Assays

Serum samples were collected by retro-orbital bleeding of the mice and obtained the day before DSS treatment (Day −1) and after DSS treatment (Days 2, 5, and 8). Supernatants from the colon explants were also analyzed after 24 h of incubation by ELISA. For IL-6 detection, capture mAb (Cat No. 504502, BioLegend) and detection mAb (Cat No. 504602, BioLegend) were used following the manufacturer’s protocol. IL-1β levels were measured using antibodies specific for the mouse cytokine (capture mAb: Cat No. 503502 and detection mAb: Cat No. 515801, BioLegend), following the manufacturer’s protocol. Samples were measured on an Autobio PHOmo microplate reader (Zhengzhou, China) in a blinded manner.

### 2.9. Macrophage Polarization

Animals were euthanized, and the femora were surgically removed and cleaned from adjacent tissues in aseptic conditions. Bone marrow cells were isolated, and 2.5 × 10^5^ cells per well were cultured for 7 days in DMEM supplemented with 10% heated-inactivated FBS plus penicillin/streptomycin in the presence of 10 ng of recombinant mouse M-CSF (Cat No. 576404, BioLegend). After 7 days, cultures contained >95% macrophages as assessed by CD11b and F4/80 staining. Bone marrow-derived macrophages (BMDMs) were then polarized to M0 (complete medium alone), M1 (100 ng/mL INFγ (Cat No. 575304, BioLegend) and 20 ng/mL LPS), or M2 (20 ng/mL IL-4 (Cat No. 574304, BioLegend)) for 24 h in the presence of different concentrations of YM or vehicle. Macrophage polarization was assessed by flow cytometry. M1 macrophages were defined as F4/80^+^CD11b^+^CD11c^+^CD206^−^ or F4/80^+^CD11b^+^CD11c^+^CD301^−^, and M2 macrophages were defined as F4/80^+^CD11b^+^CD11c^−^CD206^+^ or F4/80^+^CD11b^+^CD11c^−^CD301^+^ as previously described [41,44,45].

### 2.10. Flow Cytometry

Cells were stained in PBS containing 2% (*w*/*v*) BSA. Briefly, 7AAD (Cat No. 420403, BioLegend) was used for dead-cell exclusion. The following fluorescent conjugate-labelled antibodies were used: anti-F4/80 (clone BM8.1); anti-CD16/CD32 (clone 2.4G2); and anti-CD11b (clone M1/70) from TONBO; anti-CD301 (clone LOM-14); anti-CD206 (clone C068C2); anti-CD11c (clone N418); and anti-CD86 (clone GL-1) from BioLegend. Samples were analyzed by flow cytometry using a BD FACSCanto II instrument (BD Bioscience, San Jose, CA, USA), and data were analyzed using FlowJo version X.0.7 (Tree Star, Inc., Ashland, OR, USA).

### 2.11. Microbiota Analysis

Fecal samples were collected at 0, 7, and 14 days after YM or vehicle administration, homogenized with a porcelain mortar, filtered, and stored in glycerol buffer (10%) in PBS at −80 °C until use. A 100 mg stool sample from each mouse was used for microbial DNA isolation by utilizing the GenElute Stool DNA Isolation Kit (Sigma, St. Louis, MO, USA). The abundance of specific intestinal bacterial groups was measured by qPCR as previously described [46], with some modifications, using SYBR Green Real-Time PCR Master Mix (Thermo Fisher Scientific, Waltham, MA, USA), with the following group-specific primers to determine the amount of bacteria in each of the major groups (Operon Technologies, Huntsville, AL, USA): *Eubacteria* (all bacteria) (forward, 5′-ACTCCTACGGGAGGCAGCAGT-3′, reverse 5′-ATTACCGCGGCTGCTGGC-3′), *Eubacterium rectale*/*Clostridium coccoides* (forward, 5′-ACTCCTACGGGAGGCAGC-3′, reverse 5′-GCTTCTTAGTCAGGTACCGTCAT-3′), *Lactobacillus*/*Lactococcus* (forward, 5′-AGCAGTAGGGAATCTTCCA-3′, reverse 5′-CACCGCTACACATGGAG-3′), *Segmented filamentous bacteria* (SFB), (forward, 5′-GACGCTGAGGCATGAGAGCAT-3′, reverse 5′-GACGGCACGGATTGTTATTCA-3′), *Enterobacteriaceae* (forward, 5′-GTGCCAGCMGCCGCGGTAA-3′, reverse 5′-GCCTCAAGGGCACAACCTCCAAG-3′), *Bacteroides* (forward, 5′-GGTTCTGAGAGGAGGTCCC-3′, reverse 5′-GCTGCCTCCCGTAGGAGT -3′), and *Clostridium perfringens* (forward, 5′-CGCATAACGTTGAAAGATGG-3′, reverse 5′-CCTTGGTAGGCCGTTACCC-3′). The real-time PCR program for *Eubacteria* (all bacteria), *Eubacterium rectale*/*Clostridium coccoides*, and *Bacteroides* started with an initial step at 95 °C for 3 min, followed by 40 cycles of 10 s at 95 °C and 45 s at 63 °C. For *Lactobacillus*/*Lactococcus*, *Enterobacteriacea*, SFB, and *Clostridium perfringens* reactions, the real-time PCR program started with an initial step at 95 °C for 10 min, followed by 40 cycles of 15 s at 95 °C, 15 s at 52 °C, and 30 s at 72 °C. The conserved 16S rRNA-specific primer pair UniF340 and UniR514 was used to determine the total amount of commensal bacteria in each sample. Samples were analyzed in duplicate, and the results are expressed as relative abundance compared to the vehicle group at Day 0.

### 2.12. Statistical Analysis

Statistical analysis was performed using Prism (GraphPad 8 Software, La Jolla, CA, USA). The data are expressed as the means ± standard deviation of the mean (SD). Data distributions were tested for normality using the Shapiro–Wilk normality test (https://www.statskingdom.com/320ShapiroWilk.html, accessed on 12 December 2022). The significant differences between different data were calculated by unpaired two-tailed *t*-test (for two groups) or one-way ANOVA (for more than two groups), followed by Tukey’s multiple-comparison test. Survival curves were compared using the Log-rank (Mantel–Cox) test. The overall *p* value < 0.05 was considered statistically significant; * *p* ≤ 0.05, ** *p* ≤ 0.01, *** *p* ≤ 0.001, and **** *p* ≤ 0.0001.

## 3. Results

### 3.1. YM Supplementation Attenuates Colitis Symptoms and Improves Survival in the DSS-Induced Colitis Mouse Model

YM consumption has been shown to improve metabolic disturbances in a diabetes rat model [47], but the potential beneficial effect of YM administration in IBD, specifically in the ulcerative colitis model, has not yet been addressed. Thus, we evaluated the effect of YM administration in the DSS-induced acute colitis model, characterized by a strong intestinal inflammation, especially in the colon, with symptoms resembling those observed in human ulcerative colitis [12,48]. The choice of this model over another model of chronic colitis was based on its aggressiveness, in order to evaluate whether YM in this highly inflammatory context could reduce symptoms and inflammation. The YM solution (0.025 g per mouse) and vehicle were administrated via oral gavage 7 days before the DSS treatment started, and the administration of YM continued during the entire experiment (Figure 1A). While weight loss or colon shortening were unchanged with YM treatment, we observed reduced rectal bleeding and improved stool consistency in animals treated with YM when compared to vehicle after DSS-induced colitis (Figure 1B–D and Supplementary Figure S2). In fact, the DAI score, which considers weight loss, stool consistency, and rectal bleeding, was reduced in YM-treated mice compared to the vehicle group (Figure 1E). Hematoxylin–eosin staining in paraffin-embedded colon tissue sections showed reduced ulceration, epithelial damage, and neutrophil infiltration in the colon from YM-treated mice (Figure 1F). Accordingly, histological score analysis showed that DSS-treated mice in the vehicle group presented higher histopathological scores on Day 8 compared to the YM group (Figure 1G). These results were consistent with normal locomotion and reduced pain signals in the animals treated with YM (Supplementary Video S1). Accordingly, overall survival dramatically improved in animals receiving YM (Figure 1H). These results suggest that YM consumption reduces DSS-induced colitis symptoms, which positively impacts the survival of the animals.

### 3.2. YM Reduces Local and Systemic Inflammation Triggered by DSS Administration

**Figure 2:**
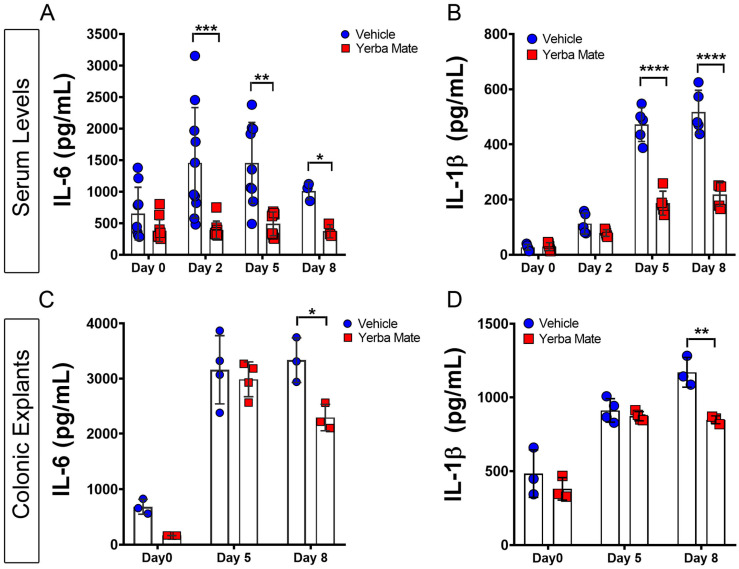
Yerba Mate reduces systemic and local inflammation triggered by DSS administration. Serum levels of IL-6 (**A**) and IL-1β (**B**) from both groups were evaluated before (Day 0) and on different days (Days 2, 5, and 8) after DSS treatment, determined by ELISA. (**C**,**D**) Time course of local IL-6 (**C**) and IL-1β (**D**) levels in colonic tissue explants after DSS treatment, quantified by ELISA assay. Data are expressed as mean ± SD from at least 5 mice per group (serum) or 3 mice per group (colon explants). * *p* < 0.05; ** *p* < 0.01; *** *p* < 0.001; **** *p* < 0.0001 by one-way ANOVA, followed by Tukey’s post test.

We next decided to analyze whether YM could reduce colonic and systemic inflammation triggered by DSS. YM consumption dramatically reduced serum levels of IL-6 and IL-1β starting on Day 2, and this trend continued during the entire DSS treatment (Figure 2A,B). For local inflammation, proinflammatory cytokines released from colonic explant cultures of YM and vehicle-treated mice harvested on different days after DSS treatment were evaluated by ELISA. Although we did not observe differences in the levels of IL-6 or IL-1β production on Day 5, we observed significant differences on Day 8, suggesting that local inflammation was also reduced by YM treatment (Figure 2C,D). These results suggest that local and systemic inflammation triggered by DSS is reduced by YM administration.

### 3.3. Increased M2 Macrophage Infiltration in Colon from YM-Treated Mice

**Figure 3:**
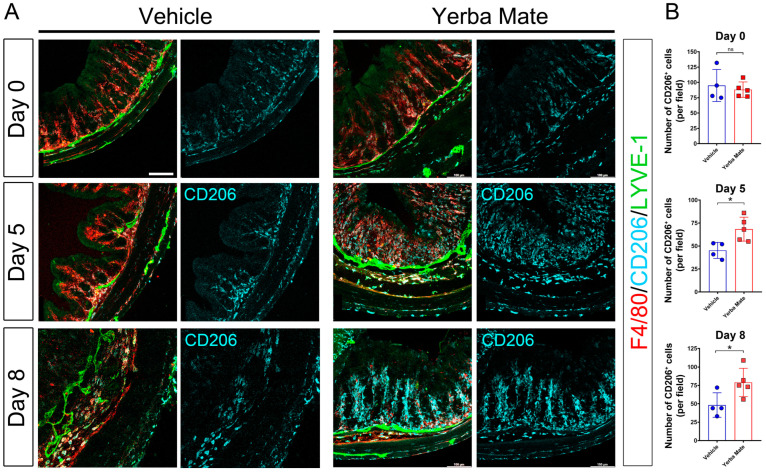
Increased M2 macrophages in the colon from Yerba Mate-treated mice: (**A**) Representative colon cryosections immunostained with specific antibodies for macrophages F4/80, CD206, and lymphatic vasculature with LYVE-1 of animals from the vehicle (left panels) or YM (right panels) groups, on Days 0, 5, and 8 after DSS treatment. The images were acquired in a Confocal microscope at 20× magnification. (**B**) CD206 positive cells were quantified after performing a z-stack and maximum projection covering the different layers of the colon sections (mucosa, submucosa, and muscularis). Data were pooled from two (n = 4–5 mice per group) independent experiments. Error bars indicate SD; n.s., not significant; * *p* < 0.05 by two-tailed Student’s *t*-test. Scale bar is 100 µm.

Because YM reduced proinflammatory cytokine production at the colon level, we next evaluated the immune cell type responsible for this effect. We focused on the macrophage population since they play an active role in the promotion of inflammation and tissue damage [49]. Moreover, since we have previously shown colonic lymphatic alterations in mice treated with DSS [41], we also wanted to evaluate whether YM could impact the architecture of the lymphatic vasculature by using the LYVE-1 antibody. Our results showed that after DSS treatment, there was an increased number of total macrophages (F4/80^+^) in both YM and vehicle control animals on the different days analyzed (Figure 3A,B). Additionally, in the submucosa, the tissue presented significant edema after DSS treatment, which was accompanied by an increase in the lymphatic vasculature (LYVE-1^+^) in both groups on Day 5 and Day 8 after DSS treatment (Figure 3A,B). When we dissected the type of infiltrating macrophage in the colon of DSS-treated mice, we found that YM-treated mice had increased numbers of anti-inflammatory M2 macrophages (CD206^+^) close to lymphatic structures, which spread to the intestinal mucosa and submucosa (Figure 3A,B), suggesting that the reduction in inflammation in the colon of YM-treated mice is not associated with changes in the lymphatic vasculature but rather could be in part explained by the increased infiltration of this anti-inflammatory macrophage subpopulation.

### 3.4. YM Administration Promotes M2 Macrophage Polarization via an Indirect Mechanism

**Figure 4:**
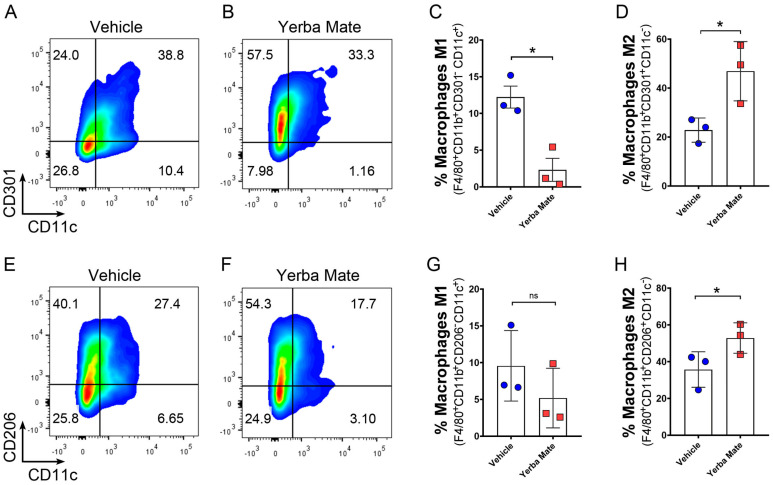
Increased M2 macrophage polarization after treatment with Yerba Mate in vivo. Animals receiving vehicle (**A**,**E**) or YM (**B**,**F**) (0.025 g per mouse) for 7 days were sacrificed, and peripheral lymph nodes were obtained and stained for M1/M2 macrophages. Two sets of markers were used to define M1/M2 macrophage populations. M1 macrophages were defined as F4/80^+^CD11b^+^CD301^−^CD11c^+^ (**C**) or F4/80^+^CD11b^+^CD206^−^CD11c^+^ (**G**), while M2 macrophages were defined as F4/80^+^CD11b^+^CD301^+^CD11c^−^ (**D**) or F4/80^+^CD11b^+^CD206^+^CD11c^−^ (**H**). Blue circles: vehicle; red squares: yerba mate. Quantification of 3 animals per group is shown. Error bars indicate SD; n.s., not significant; * *p* < 0.05 by unpaired *t*-test.

**Figure 5:**
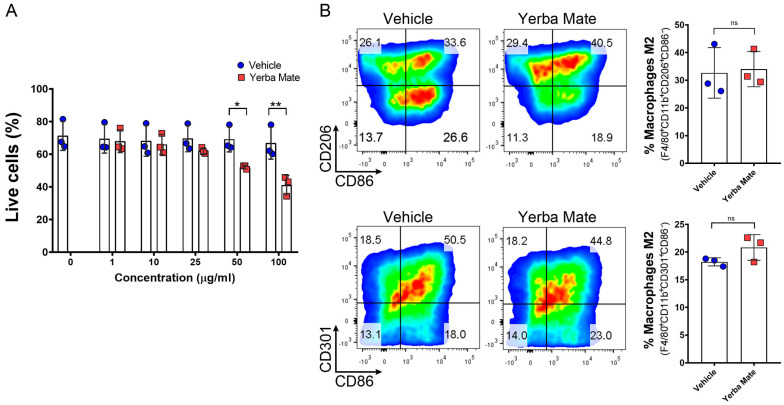
Yerba Mate indirectly promotes M2 macrophage polarization. BMDMs were obtained from *WT* mice and cultured under M2 polarization in the presence of YM or vehicle at different concentrations for 24 h. (**A**) Cell survival measured by 7-AAD at different YM concentrations. (**B**) Representative density plots (left) and quantification (right) of M2 macrophages defined as F4/80^+^CD11b^+^CD206^+^CD86^−^ (upper panels) or F4/80^+^CD11b^+^CD301^+^CD86^−^ (lower panels) analyzed by FACS. Data are expressed as means ± SD from 3 independent experiments; n.s., not significant; * *p* < 0.05; ** *p* < 0.01 by one-way ANOVA followed by Tukey’s post test.

As an increased number of anti-inflammatory M2 macrophages were observed in the colon of YM-treated mice after DSS treatment, we hypothesized that YM could directly promote M2 macrophage polarization in vivo. Thus, we treated mice with YM or vehicle for 7 days, and macrophage polarization was evaluated in peripheral lymph nodes by FACS. Our results showed stronger M2 polarization, defined as F4/80^+^CD11b^+^CD11c^−^CD206^+^ or F4/80^+^CD11b^+^CD11c^−^CD301^+^ in animals treated with YM when compared to vehicle controls (Figure 4A–H). We next decided to evaluate whether YM can directly promote M2 macrophage polarization in vitro by adding YM or vehicle to bone marrow-derived macrophages (BMDMs) in an M2 polarization condition. First, we checked the effect of different concentrations of YM on BMDM viability and found that 50 and 100 µg/mL of YM solution significantly reduced cell survival (Figure 5A). Thus, we decided to use 25 µg/mL of YM solution in our in vitro experiments. Interestingly, at this concentration, we did not observe any effect of YM over M2 macrophage polarization in vitro, suggesting that YM did not directly promote anti-inflammatory macrophage polarization (Figure 5B). In line with these observations, the analysis of intestinal microbiota from animals receiving YM or vehicle showed an overall reduction in the total bacterial content in the YM group 14 days after the start of treatment (Supplementary Figure S3). This result is mainly explained by the reduction in the *Eubacterium rectale*/*Clostridium coccoides* and *Enterobacteriaceae* groups (Supplementary Figure S3). Interestingly, a marked increase in the *Lactobacillus*/*Lactococcus* group was evident in the animals receiving YM on Days 7 and 14 after the start of treatment (Supplementary Figure S3). All these results suggest that changes in the intestinal microbiota content promoted by YM treatment could be in part responsible for M2 macrophage polarization in vivo, which may in turn reduce inflammation and colitis symptoms in the DSS-induced colitis model.

## 4. Discussion

In this work, we investigated the effect of YM on colitis symptoms and macrophage polarization. Our study demonstrates that YM consumption reduces DSS-induced colitis symptoms and improves survival in part by increasing the infiltration of M2 macrophages in the colon with the concomitant reduction in local and systemic inflammation. Moreover, YM changed gut microbiota composition, reducing *Eubacterium rectale*/*Clostridium coccoides* and *Enterobacteriaceae* groups and increasing the *Lactobacillus*/*Lactococcus* group, suggesting that changes in gut microbiota by YM could promote M2 macrophage polarization.

The use of medicinal plants for the treatment of IBD, particularly ulcerative colitis, has been growing worldwide during the last few decades, despite the limited evidence and lack of knowledge about the mechanism of action of several of the plants used [50]. Accordingly, recent efforts have been focused on the analysis of different bioactive compounds found in medicinal plants and their therapeutic targets [51]. It has been found that polyphenols from medicinal plants reduce DSS-induced colitis symptoms by decreasing inflammatory cytokines, such as IL-1β and IL-6, together with antioxidant capacity [52]. Interestingly, our chemical analysis of YM revealed that chlorogenic acid, an important biologically active dietary polyphenol, was the highest bioactive compound in our extract (2461 ± 122 mg/Kg). In fact, it has been recently shown that chlorogenic acid reduces type 1 diabetes-induced inflammation in a rat model of the disease [53]. Mechanistically, chlorogenic acid has different targets: It can inhibit different signaling pathways such as nuclear factor-κB (NF-κB) or p38-mitogen-activated protein kinase (MAPK) signaling pathways; it can directly inhibit the production of proinflammatory cytokines such as IL-6 or TNFα; and it can also inhibit cyclooxygenase-2 (COX-2) among other targets (reviewed in [54]). In fact, chlorogenic acid could directly promote in vivo M2 macrophage polarization in a mouse model of pneumonia [55]. Moreover, it has already been demonstrated that chlorogenic acid can directly modulate the gut microbiota and reduce inflammation [56,57]. Since we observed an increased number of M2 macrophages in the colon of DSS-treated animals supplemented with YM, we can speculate that the observed protection of YM could be in part explained by the high content of chlorogenic acid in our extract. However, more studies are necessary to confirm this hypothesis.

Changes in gut microbiota have been observed in ulcerative colitis patients and DSS-treated mice, and these changes have been related to increased inflammation [58,59]. For example, *Eubacterium rectale*, a Gram-positive bacterium, has been shown to increase inflammatory damage in the DSS-induced colitis mouse model [60]. The *Enterobacteriaceae* group, comprising rod-shaped Gram-negative bacteria that include several pathogens such as *Klebsiella*, *Enterobacter*, or *Salmonella*, is enriched in the gut microbiome of IBD patients and can modulate colitis [61,62]. On the other hand, some beneficial bacteria can improve colitis symptoms. This is the case with the *Lactobacillus*/*Lactococcus* group, where several members can alleviate DSS-induced colitis symptoms [63]. Our observation that YM consumption reduced *Eubacterium rectale*/*Clostridium coccoides* and *Enterobacteriaceae* groups and increased the *Lactobacillus*/*Lactococcus* group could in part explain the beneficial properties of the medicinal plant since controlling dysbiosis and stimulating the presence of favorable bacteria could improve colitis symptoms [64]. Moreover, a relationship between the consumption of polyphenols and an increase in beneficial microbiota has been well described [65]. Furthermore, several studies have shown that YM can modulate microbiota [66,67]. Since YM seems to work not directly toward macrophage polarization but by favoring changes in the microenvironment, we can speculate that polyphenols, particularly phenolic acid, promote M2 macrophage polarization and reduce inflammation by modulating gut microbiota. Further studies are necessary to dissect the specific mechanism of M2 polarization by YM. 

Because of the importance of the M1/M2 macrophage polarization ratio in colitis, it has been proposed that molecules or drugs that increase M2 or reduce M1 polarization could protect against DSS-induced colitis and therefore have the potential to be a therapeutic strategy to treat this disease [20,21,22]. In fact, the use of medicinal herbs targeting macrophage polarization has been shown to reduce colitis in murine models. For example, the active extract of ginseng (a plant used in traditional Chinese medicine) has been shown to reduce colitis symptoms by decreasing the levels of IL-1β and TNFα and promoting M2 macrophage polarization [21,68]. Another medicinal plant with anti-inflammatory effects is *Ilex kudingcha* from the genus Ilex, the same genus as that of *Ilex paraguariensis*, which is used as a traditional Chinese tea, and this plant has been recently shown to improve DSS-induced colitis and inflammation [69]. The fact that YM also reduces colitis symptoms and inflammation by promoting M2 macrophage polarization suggests its potential therapeutic use for IBD patients. However, the fact that YM only promotes M2 macrophage polarization in vivo but not directly in BMDMs in vitro suggests that YM does not directly affect macrophage polarization but affects the microenvironment to promote M2 macrophage polarization. Additional studies are necessary to evaluate the specific cellular and molecular immunomodulatory pathways modulated by YM.

It is important to highlight that besides the low prevalence of IBD in South American countries, IBD patients in this region develop a less aggressive phenotype and a more limited disease extension, despite the reduced use of biological therapies [70]. Although there are several differences in genetics, diets, and lifestyle among the different regions, we can speculate that YM consumption in Andean countries may contribute to the reduced incidence observed in this region. Future studies are necessary to explain the different factors contributing to the low prevalence of IBD in South American countries.

The strength of our study lies in the consistency of our results, together with the different techniques used to show the protective effect of YM over colitis and the effect of YM over the M2 macrophage population. The number of animals used and independent repetitions in vivo are also strengths of our work. Our study has also some limitations. First, our study focused on the analysis of the proinflammatory cytokines IL-1β and IL-6, but we did not evaluate TNFα, one of the main cytokines involved in the pathogenesis of IBD [71]. Second, even though we analyzed the expression of different M2 markers, we did not measure the levels of anti-inflammatory cytokines such as IL-10, a cytokine produced by anti-inflammatory macrophages capable of contributing to their immunosuppressive phenotype [72,73]. Third, although we observed that the prophylactic administration of YM reduced colitis symptoms, the therapeutic administration of YM was not evaluated. For future research, it would be beneficial to investigate the effect of specific bioactive compounds of YM on macrophage polarization, together with the molecular pathways modulated by YM. The relationship between YM–gut microbiota and macrophage polarization in the DSS-induced colitis model should be also addressed. Additionally, further studies are warranted to directly evaluate the effect of YM administration in IBD patients.

## 5. Conclusions

In summary, in this work, we reported that YM consumption reduces symptoms and improves survival in a DSS-induced colitis model in mice. Reduced inflammation and increased M2 macrophage infiltration in the colon were observed in the animals treated with YM. Changes in gut microbiota were also observed in animals receiving YM. Accordingly, YM promoted M2 macrophage polarization in vivo but not in vitro, suggesting that this medicinal herb, through an indirect mechanism that could involve changes in the intestinal microbiota, contributes to skewing the M1/M2 ratio toward an anti-inflammatory phenotype.

## Data Availability

All data are presented in the manuscript and are available upon request due to due to privacy reasons.

## Supplementary Materials

The following supporting information can be downloaded at: https://www.mdpi.com/article/10.3390/nu16111616/s1, Figure S1: Analysis of Yerba Mate components by HPLC.; Figure S2: Colon length in DSS-treated animals; Figure S3: Changes in gut microbiota induced by Yerba Mate consumption. Video S1: Reduced lethargy in animals receiving Yerba Mate”, we removed the caption, please confirm.
