# Supplementary material for: Yerba Mate (Ilex paraguariensis) Reduces Colitis Severity by Promoting Anti-Inflammatory Macrophage Polarization
Source: Nutrients. 2024 May 25;16(11):1616. doi: 10.3390/nu16111616 (PMC11174081; doi:10.3390/nu16111616)
Supplement: Supplementary file 1 [file nutrients-16-01616-s001.zip › supplementary/nutrients-2994305-supplementary.pdf]

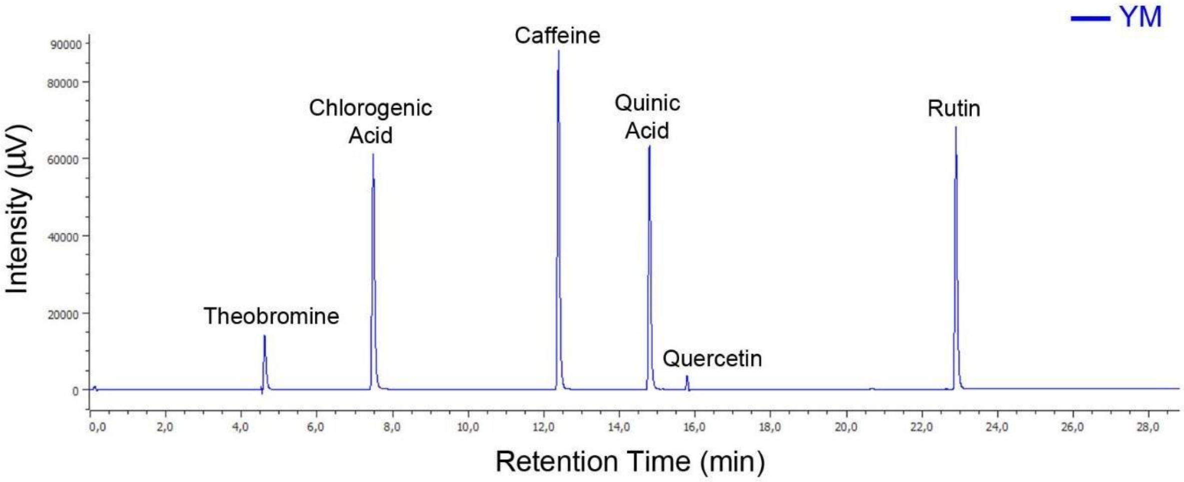


| Analyte | Concentration (mg/Kg) |
|---|---|
| Chlorogenic Acid | 2461 ± 122 mg/Kg |
| Caffeine | 2388 ± 189 mg/Kg |
| Theobromine | 892 ± 55 mg/Kg |
| Rutin | 486 ± 16 mg/Kg |
| Quercetin | 39 ± 7 mg/Kg |
| Quinic Acid | 18 ± 4 mg/Kg |

**Supplementary Figure S1:** Analysis of yerba mate components by HPLC.

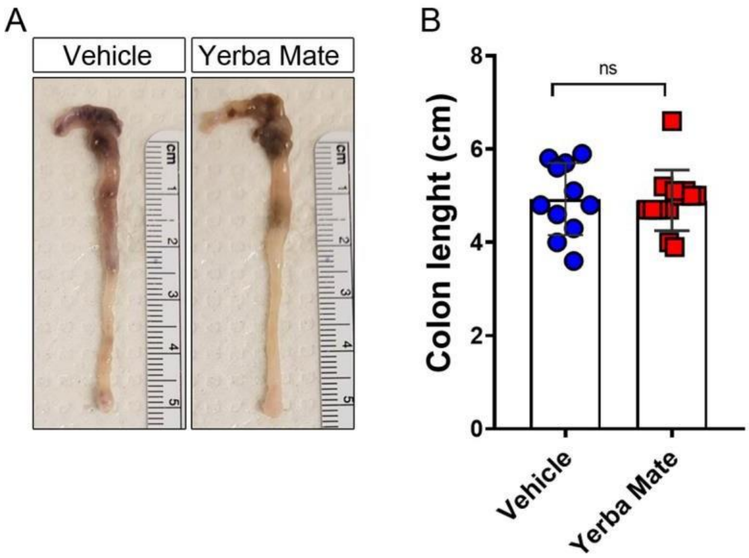


**Supplementary Figure S2:** Colon length in DSS-treated animals

(A) Macroscopic appearance and (B) quantification of the length of colons at day 8 of *WT* mice receiving YM or vehicle. Data are plotted as means ± SD from 11 (vehicle group) and 15 (YM group) mice from three independent experiments. ns = non-significant by Unpaired t-test

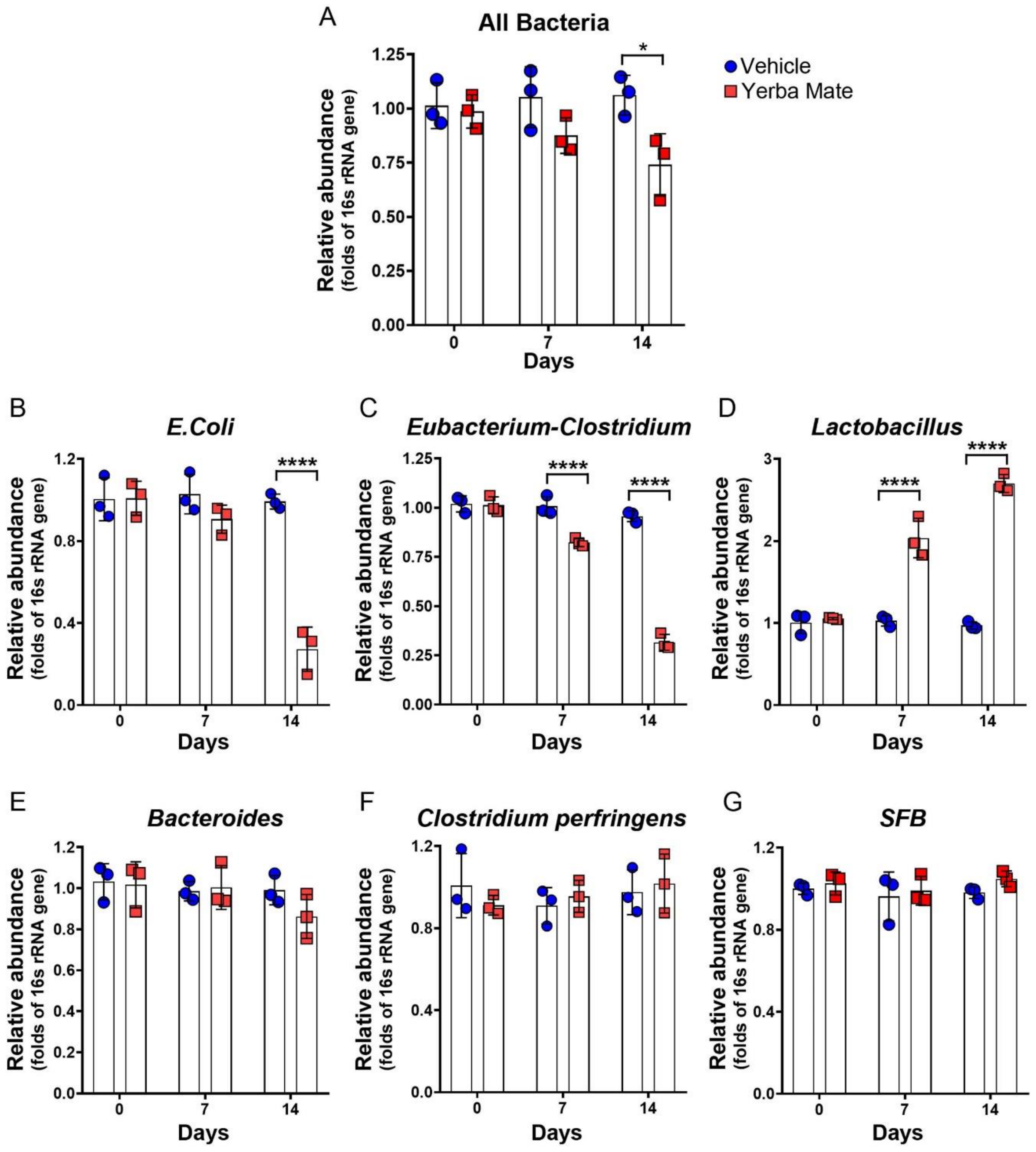


**Supplementary Figure S3:** The relative abundance of fecal Eubacteria (all bacteria)(A), *Enterobacteriaceae* (*E.Coli*) (B), *Eubacterium rectale*/*Clostridium coccoides* (C), *Lactobacillus*/*Lactococcus* (*Lactobacillus*) (D), *Bacteroides* (E), *Clostridium perfringens* (F), and *Segmented filamentous bacteria groups* (*SFB*) (G), from animals receiving YM or vehicle at day 0, 7 and 14 days was evaluated by real-time PCR. The conserved 16S rRNA-specific primer pair UniF340 and UniR514 was used to determine the total amount of commensal bacteria in each sample. * $P < 0.05$; **** $P < 0.0001$ by One-way ANOVA followed by Tukey's post-test.
